# Recently enlisted patients in general practice use more health care resources

**DOI:** 10.1186/1471-2296-8-64

**Published:** 2007-11-29

**Authors:** Lea Jabaaij, Dinny H de Bakker, Henk J Schers, Patrick JE Bindels, Janny H Dekker, François G Schellevis

**Affiliations:** 1 NIVEL (Netherlands Institute for Health Services Research), PO Box 1568, 3500 BN Utrecht, The Netherlands; 2 Department of General Practice, University Medical Centre St Radboud Nijmegen, The Netherlands; 3 Department of General Practice, Academisch Medisch Centrum (AMC), University of Amsterdam, The Netherlands; 4 Department of General Practice, University Medical Centre, Groningen, The Netherlands; 5 Department of General Practice, Vrije Universiteit (VU) University Medical Centre, Amsterdam, The Netherlands

## Abstract

**Background:**

The continuity of care is one of the cornerstones of general practice. General practitioners find personal relationships with their patients important as they enable them to provide a higher quality of care. A long-lasting relationship with patients is assumed to be a prior condition for attaining this high quality. We studied the differences in use of care between recently enlisted patients and those patients who have been enlisted for a longer period.

**Methods:**

104 general practices in the Netherlands participated the study. We performed a retrospective cohort study in which patients who have been enlisted for less than 1 year (n = 10,102) were matched for age, sex and health insurance with patients who have been enlisted for longer in the same general practice. The two cohorts were compared with regard to the number of contacts with the general practice, diagnoses, rate of prescribing, and the referral rate in a year. These variables were chosen as indicators of differences in the use of care.

**Results:**

In the year following their enlistment, a higher percentage of recently enlisted patients had at least one contact with the practice, received a prescription or was referred. They also had a higher probability of receiving a prescription for an antibiotic. Furthermore, they had a higher mean number of contacts and referrals, but not a higher mean number of prescriptions.

**Conclusion:**

Recently enlisted patients used more health care resources in the first year after their enlistment compared to patients enlisted longer. This could not be explained by differences in health.

## Background

The continuity of care is one of the cornerstones of general practice in the Netherlands. The patient is known by the general practitioner who cares for him, or her, over a prolonged period. This continuity of care is valued by patients [1-3] and enhances the work satisfaction of general practitioners [4]. However a stable and long-lasting relationship with patients is assumed to be a prior condition for this continuity of care. But general practitioners do not have a long-lasting relationship with all their patients. The listed practice population is changing continuously, because patients die or move. Patients might also change their GP for reasons of dissatisfaction with the previous GP or retirement of their GP.

In the Netherlands the average turnover rate, calculated by the number of newly registered patients *plus *the number of patients leaving the practice, was found to be 12% of the practice population per year with a peak of 16% in highly urbanized areas [5]. As a consequence, general practitioners have to invest again and again in new relationships. This might influence their workload, especially in practices with a high turnover rate and complex patient populations. Hjortdahl studied how the duration of relationships between general practitioners and patients influenced use of health care resources. General practitioners stated that 'knowing the patient' saved them time in consultations [6]. General practitioners' own, subjectively evaluated, knowledge about the patient's medical history was found to be helpful in deciding on therapeutic actions [7]. And important: patients with a longer relationship with their general practitioner were slightly more satisfied with the consultations [8]. However, the relationship between 'knowing the patient' and use of health care resources was not simple. Prior knowledge influenced the care in two directions. Doctors with prior knowledge were often more liberal with prescriptions and one in six consultations was prolonged because of a social conversation and problems not related to an illness. Also, effects differed for new and chronic conditions.

Hjortdahl operationalized continuity of care as the duration of the relationship between the doctor and the patient, using a subjective statement: 'knowing the patient'. In our study we used a more objective measure: duration of enlistment. In this article we answered the following question: do recently enlisted patients use more or less health care resources, as delivered by general practitioners, compared to patients enlisted for a longer period? All patients enlisted in a general practice over the previous year were matched with patients enlisted in the same practice but for a longer period. We compared these two groups with respect to consultation rates, diagnoses, prescribing of drugs and referral to other health care providers.

## Methods

### Practices

Data were retrieved from the electronic medical records (EMR) in 104 general practices employing 195 general practitioners with 400,000 enlisted patients, participating in the Second Dutch National Survey of General Practice (DNSGP-2) in 2001. 53% of the practices was a solo practice, 23% a duo practice and 24% a group practice (mean number of GPs is 3.6 [9,10]). These provide a representative sample of Dutch general practices.

### Patients

We performed a retrospective cohort study in which two cohorts were constituted. All patients over two years old and newly enlisted in 2001 were included in the analyses (n = 10,102). Data were used on the 12-month period following their addition to the list. For every patient included, a control patient was selected from the patient list within the same practice, matched for age in five year intervals, gender, and type of health care insurance. In 2001 health care insurance in the Netherlands could be either public or private depending on income and therefore we used this as a proxy for socio-economic status (SES), that is low and medium as opposed to high. For the matched control group data were used on the same time interval as the patient he or she, was matched to. Patients' mean age was 31.4 years, 81% was younger than 44 years (national population: 62%), 48.5% was male and 38.3% was of high SES (national population: 33.5%). Recently enlisted patients (and their matched controls) were younger and of higher SES [11]. The median enlistment period of the control group was five years.

### Health care resources

The EMR encompasses routinely registered data on contacts with the general practice, morbidity, referrals to other health care providers and drugs prescribed for every patient enlisted in the practice. The consultation rate was defined as the number of face-to-face contacts during a period of twelve months. Health problems were coded by the general practitioner, using the International Classification of Primary Care (ICPC) [12]. Prescriptions were coded according to the Anatomical Therapeutic Chemical classification system (ATC). We calculated the mean number of prescriptions in a year per patient – that is the total prescription rate – and the mean number of prescriptions for antibiotics. The total prescription rates might be biased by a high number of repeat prescriptions in patients enlisted over a longer period. Because antibiotics are not prescribed routinely, and therefore their use has to be monitored conscientiously, these prescriptions might add more information on the differences in the rates of prescribing between recently, and longer enlisted, patients.

In the Netherlands the general practitioner functions as the 'gatekeeper' of care, meaning that patients need a referral for specialist health care or for other primary health care workers [13]. The number of referrals can be seen as an indication of the ability of general practitioners to deal with requests for treatment themselves. We calculated the mean number of new referrals per patient.

We included data on 81,146 face-to-face contacts with the general practice, 94,679 prescriptions, 4,789 prescriptions for antibiotics, and 6,329 referrals.

### Analyses

Using Chi-square tests, we calculated the probability for each patient of having a face-to-face contact with the general practice, receiving a prescription or being referred. We also calculated mean figures for the number of contacts with the general practice, prescriptions and referrals, and tested for significance between the two groups using univariate variance analyses (Student's t-test). Because of our large sample size, we settled on p <. 01 for significance.

## Results

### Chronic diseases

Recently enlisted patients and their matched controls were not found to differ in prevalence of chronic diseases. The number of patients suffering from diabetes mellitus (ICPC code T90), hypertension (K85, K86, K87), astma/COPD (R95, R96), coronary heart disease (K74, K75, K76, K77) or depression (P03, P76) did not differ significantly between the two groups.

### Consultation rate

A higher percentage of recently enlisted patients had at least one contact with the general practice within a year, compared to patients enlisted over a longer period (see Table 1). As a group, they also had a higher mean number of contacts.

**Table 1:** Utilization of health care resources in general practice (GP) for recently and longer enlisted patients

	Recently (< 1 year)	Longer (> 1 year)	p
	(N = 10,102)	(N = 10,102)	
Contacts with GP			
- Patients with 1 or more contacts with GP (%)	77.4	60.9	*
- Mean number of contacts in one year (95% CI)	3.23 (3.15; 3.31)	2.84 (2.76; 2.93)	*
Prescriptions			
- Patients with 1 or more prescriptions (%)	66.7	57.5	*
- Mean number of prescriptions in one year (95% CI)	4.68 (4.46; 4.92)	4.69 (4.48; 4.87)	n.s.
Referrals			
- Patients with 1 or more new referrals (%)	20.4	17.5	*
- Mean number of new referrals in one year (95% CI)	0.27 (.26; .28)	0.24 (.23; .26)	*

* = p < .001, n.s. = not significant, Chi-square and t-test

In the top-10 of diagnoses registered during the first contact (for those patients who attended), 'oral contraceptive' ranks number one, followed by 'no disease' and 'upper respiratory tract infection' (see Table 2). Recently enlisted patients have significantly higher odds ratios for 'no disease'. The number of recently enlisted patients for whom the general practitioner did not register a diagnosis is also higher than for the controls. The chance of presenting with an upper respiratory tract infection or an uncomplicated hypertension is lower for recently enlisted patients.

**Table 2:** Top-10 of diagnoses registered during the first contact by the general practitioner

		Odds ratio	95% C.I.	Per 1000 patients
	no diagnosis	1.5*	1.28; 1.68	66.6

1	W11 'oral contraceptive'	1.1	.87; 1.27	31.9
2	A97 'no disease'	4.4*	3.37; 5.86	27.8
3	R74 'upper respiratory tract infection'	0.7*	.60; .91	25.6
4	S74 'dermatophytosis'	1.1	.85; 1.35	21.8
5	L03 'low back pain'	1.0	.74; 1.23	17.3
6	S88 'contact dermatitis, other eczema'	0.8	.59; 1.00	16.8
7	K86 'uncomplicated hypertension'	0.7*	.56; .94	16.5
8	A04 'general weakness'	1.0	.78;1.34	16.0
9	R05 'cough'	0.8	.61; 1.05	15.3
10	U71 'cystitis'	1.0	.76; 1.34	14.2

Only patients who attended in one year are presented (odds ratios and per 1000 patients who attended). Odds ratios for recently enlisted patients are presented with longer enlisted patients as the reference group

To exclude the possibility that the higher mean of consultation rates for recently enlisted patients is caused by introductory consultations in which 'no illness' is presented, we excluded these from the analyses. The mean number of consultations decreased from 3.23 to 3.20 per patient and remained significantly higher compared to the mean for the matched controls, who had 2.84 contacts per patient in a year.

### Prescriptions

A higher number of recently enlisted patients received a prescription, compared to patients enlisted for longer (see Table 1). The mean number of prescriptions per patient did not differ between the two groups, indicating that more recently enlisted patients had prescriptions but each of these had fewer items prescribed.

#### Antibiotics

About 15% of all patients received one or more prescriptions for a systemic antibiotic (ATC code J01) in a year. This percentage was slightly but significantly higher for recently enlisted patients (15.8%) compared to their matched controls (13.8%). The mean number of antibiotic prescriptions did not differ between the two groups (data not shown). Furthermore, we observed no differences in the diagnoses for which antibiotics were prescribed (data not shown).

### Referrals

Recently enlisted patients had a higher probability of being referred to a primary or secondary care professional. Also, the mean number of referrals was higher for recently enlisted patients (see Table 1). This pattern did not differ between the various primary and secondary care professionals (data not shown).

## Discussion

We found that a larger proportion of recently enlisted patients had at least one contact, a referral and/or a prescription, including antibiotics, within a year, compared to patients enlisted for longer. This difference not only concerns the proportion of patients, but also the mean numbers of actions, as recently enlisted patients had more contacts in a year and slightly more referrals. The mean number of prescriptions, including antibiotic prescriptions, did not differ between the two groups. While not all differences are that large, they do all point in the same direction: recently enlisted patients use more health care resources as delivered by general practitioners than longer enlisted patients. The results can not be explained by differences in chronic diseases.

Why the two cohorts of patients differed on 'upper respiratory tract infection' and 'uncomplicated hypertension' during the first consult is not clear. The two cohorts did not differ in chronic conditions, amongst which hypertension. Apparently, hypertensive patients ordered enough medication before moving to another GP. Subsequently, another or no complaint (an introductory consultation?) might have been the first reason to consult the doctor.

We used antibiotic prescriptions to validate the differences in prescription rates between the two groups of patients. Antibiotics are not prescribed routinely or as repeat prescriptions in contrast to some other medication. General practitioners might be less reluctant to prescribe antibiotics to newly enlisted patients to show their willingness. Or in contrast, they might be more reluctant because they do not know these patients yet. However, antibiotic prescriptions followed the same pattern for both groups as the overall prescription rate.

General practitioners registered more contacts with 'no disease' for recently enlisted patients, probably indicating an introductory consult. In general, both the patient and the general practitioner value an introductory consultation as a necessary beginning to a longer-lasting medical relationship where trust and knowing each other are important. This is in contrast with specialist care where introductory consultations, without any medical reason, are uncommon. Nevertheless, introductory consultations could not explain the higher consultation rate of recently enlisted patients. After removing them from the analyses, we still found higher consultation rates for recently enlisted patients compared to their matched controls.

Patients were matched within practices and not within general practitioners. Would results have been different if we had decided otherwise? Previous research in the Netherlands showed that variability in attitudes and decisions of physicians adapts to what is usual in the work environment under consideration, both hospital and general practice. In other words, variation in behaviour is lesser between colleagues working in the same practice than between practices [10,14,15]. Also, in this study results did not differ between the different practice organisations (data not shown).

### Changing of GP

There are numerous reasons for a patient for changing of GP. Probably the most prevalent reason is moving home. Is 'moving home' directly related to health? In a study on the motives for moving, only 3–5% of all respondents stated that health was the most important reason to move [16]. Our results gives no evidence of health related reasons to move: no differences in chronic conditions and a relatively young patient group. On the other hand, moving to another house is a stressful life event often associated with other life events like living on your own, marriage, having children and divorce. Stressful life events are known to influence health and/or help seeking behaviour [17,18]. So, stress-related events might have influenced some of the results.

Recently enlisted patients had a slightly higher probability of being referred to other health care workers. In the Netherlands, only 10 to 15 percent of all moves are more than 100 kilometres (62 miles) from the former home [16]. Two thirds of all people changing house stay within their communities. The catchment area of hospitals, including specialist care, covers a larger region than that of general practices, making it less necessary to switch to specialist care in another city. So we doubt that having moved might be the reason for the higher referral rate.

There are other reasons than moving house for changing your general practice. For example, dissatisfaction with a former general practitioner or his or her policy. The new general practitioner might be more prepared to act according the wishes of the patient, for example in prescribing or referring.

In conclusion, there are numerous reasons for a short enlistment period. And several of these reasons might have an independent effect on consumption of care, apart from the potential effect of 'provider continuity'. Because we do not know why patients changed of GP, we could not include the reasons for a short enlistment period in our analyses. But we expect that the different reasons for a short enlistment period are randomly distributed in our relatively large sample.

Several other studies found greater use of resources when general practitioners are not familiar with the patient [19,20]. Depending on the unique national (primary) health care system, researchers faced different problems in selecting patient groups. In other studies, for example (re-)enrolment into health care programmes, financing, reimbursement or free access to different general practitioners at the same time had to be taken into account before explaining differences between recently, and longer, enlisted patients. These factors did not play a role in our study. In the Netherlands, the general practitioner is gatekeeper for specialist care. The fact that 98% of the population is enlisted with a specific general practice, enabled the selection of the two cohorts of patients for this study. However, we do not know the reason why patients changed of GP and we can not exclude that other factors than 'short enlistment period' might play a role in differences between recently and longer enlisted patients.

### Knowing the patient

Hjortdahl [6] suggested that the doctor's subjective evaluation of 'knowing the patient' 'leads' to less consumption of resources. It would take on average one to five years, or four to five consultations within 12 months, for doctors to develop a moderate knowledge base of their newly enlisted patients. Freeman reported that the nature and quality of the doctor-patient relationship is more important than the number of contacts [21]. It is tempting to speculate that our findings on the differences in the use of health care resources are a consequence of the fact that doctors and patients are not familiar with each other. General practitioners have no knowledge about the context of the patient, his or her ability to cope with illness, and their past experiences with general practitioners. Patients are not familiar with the general practitioner and his or her way of acting as a general practitioner. Mutual trust has to grow.

### Implications

Do recently enlisted patients receive better care when more of them receive a prescription or a referral? Or does the general practitioner behave more defensively when treating patients for the first time? A whole array of variables influences the medical behaviour of general practitioners. They could include the frequency of the patient's visits to the general practice, the morbidity presented by the patient, the diagnostic competences of the general practitioners and the communicative skills of both the general practitioner and the patient. These variables will interact with each other, thereby influencing the outcome: a prescription or a referral. Knowledge about the patient is often essential for interpreting complaints. The familiarity between the patient and the doctor might influence one or more of the variables in the processes leading to a prescription or referral. On the other hand, familiarity can also blind both the general practitioner and patient to less obvious factors.

### The future

The data in our study originate from 2001. In the years since, some changes took place in Dutch general practice. For example, more GPs provide out of office care regionally in large GP corporations, more professionals such as nurse practitioners entered the general practice, and the gatekeeping role for physiotherapy was abrogated. These changes might have as consequence that patients see more different professionals in their general practice, thereby influencing provider continuity.

## Conclusion

Previous studies found that 'knowing the patient' influences the use of health care resources, but the effects could mean both more, or less, use of resources. This study refines previous results, using a straight forward operationalization of continuity of care: period of enlistment of the patient. Recently enlisted patients with a general practice used more health care resources in the first year after their enlistment compared to patients enlisted longer. This could not be explained by differences in chronic conditions. It is open for speculation in how far differences in the use of health care resources are influenced by factors related to 'moving house' or differences in the strength of the doctor-patient relationships.

We conclude that, in general practice, a high continuity of care leads to less use of health care resources.

## Competing interests

The author(s) declare that they have no competing interests.

## Authors' contributions

FS, PB and JD formulated the research question. FS, DdeB, HS, PB, JD and LJ participated in the design of the study. LJ and FS carried out the study. All authors were involved in interpretation of the data. LJ performed the statistical analysis and drafted and revised the manuscript. All authors critically reviewed draft versions of the manuscript. All authors read and approved the final manuscript.

## Pre-publication history

The pre-publication history for this paper can be accessed here:


